# Dramatic response of *BRAF* V600E-mutant epithelioid glioblastoma to combination therapy with BRAF and MEK inhibitor: establishment and xenograft of a cell line to predict clinical efficacy

**DOI:** 10.1186/s40478-019-0774-7

**Published:** 2019-07-25

**Authors:** Yu Kanemaru, Manabu Natsumeda, Masayasu Okada, Rie Saito, Daiki Kobayashi, Takeyoshi Eda, Jun Watanabe, Shoji Saito, Yoshihiro Tsukamoto, Makoto Oishi, Hirotake Saito, Masayuki Nagahashi, Takahiro Sasaki, Rintaro Hashizume, Hidefumi Aoyama, Toshifumi Wakai, Akiyoshi Kakita, Yukihiko Fujii

**Affiliations:** 1 From the Departments of Neurosurgery, Niigata University, 1-757 Asahimachidori, Chuo-ku, Niigata, Japan; 2 Pathology, Brain Research Institute, Niigata University, Niigata, Japan; 3 Department of Radiology and Radiation Oncology, Niigata University Graduate School of Medical and Dental Sciences, Niigata, Japan; 4 Division of Digestive and General Surgery, Niigata University Graduate School of Medical and Dental Sciences, Niigata, Japan; 5 Department of Neurosurgery, Northwestern University, Chicago, IL USA

**Keywords:** Epithelioid glioblastoma, *BRAF* V600E, Targeted therapy, Precision medicine

## Abstract

Epithelioid glioblastoma is a rare aggressive variant of glioblastoma (GBM) characterized by a dismal prognosis of about 6 months and frequent leptomeningeal dissemination. A recent study has revealed that 50% of epithelioid GBMs harbor three genetic alterations – *BRAF* V600E mutation, *TERT* promoter mutations, and homozygous deletions of *CDKN2A/2B*. Emerging evidence support the effectiveness of targeted therapies for brain tumors with *BRAF* V600E mutation. Here we describe a dramatic radiographical response to combined therapy with BRAF and MEK inhibitors in a patient with epithelioid GBM harboring *BRAF* V600E mutation, characterized by thick spinal dissemination. From relapsed tumor procured at autopsy, we established a cell line retaining the *BRAF* V600E mutation, *TERT* promoter mutation and *CDKN2A/2B* loss. Intracranial implantation of these cells into mice resulted in tumors closely resembling the original, characterized by epithelioid tumor cells and dissemination, and invasion into the perivascular spaces. We then confirmed the efficacy of treatment with BRAF and MEK inhibitor both in vitro and in vivo. Epithelioid GBM with *BRAF* V600E mutation can be considered a good treatment indication for precision medicine, and this patient-derived cell line should be useful for prediction of the tumor response and clarification of its biological characteristics.

**Electronic supplementary material:**

The online version of this article (10.1186/s40478-019-0774-7) contains supplementary material, which is available to authorized users.

## Introduction

Epithelioid glioblastoma is a rare aggressive variant of glioblastoma (GBM), newly proposed in the 2016 WHO classification, characterized by frequent leptomeningeal dissemination [1–5] and poor overall survival of approximately 6 months [2, 6]. Genetic analyses have indicated a high percentage of *BRAF* V600E (50–93%) [7, 8] and *TERT* promoter mutations (70%), and homozygous deletions of *CDKN2A/2B* (79%) [7]. In a series of 14 epithelioid GBMs, 7 cases (50%) harbored all 3 alterations [7], suggesting that epithelioid GBMs have recurrent genetic alterations, making them a prime candidate for precision-based medicine.

Progress in genetic studies and targeted therapies has vastly improved the treatment of some cancers [9, 10]. Several case reports have described the success of targeted treatment to *BRAF* V600E-mutant brain tumors such as ganglioglioma [11, 12], pleomorphic xanthoastrocytoma (PXA) [13, 14] and papillary craniopharyngioma [15, 16]. The VE-BASKET study investigating the efficacy of the BRAF inhibitor vemurafenib against *BRAF* V600E-mutant cancers demonstrated that efficacy varied by histologic subtypes in patients with *BRAF* V600E-mutant brain tumors [17], suggesting that some *BRAF* V600E-mutant tumors have multiple concurrent drivers. It is still unclear which patients would benefit from precision medicine. Thorough genetic studies and establishment of patient-derived cell lines would support decision making in relation to targeted treatment.

Here we report a case of epithelioid GBM harboring *BRAF* V600E mutation that showed a dramatic radiographical response to combined therapy with BRAF and MEK inhibitor after spinal dissemination. From a recurrent tumor sample obtained at autopsy, we established a cell line and used it to confirm both in vitro and in vivo that combination treatment was effective. Our findings suggest that targeted therapy would be beneficial for patients with epithelioid glioblastoma harboring *BRAF* V600E mutation, and that establishment of cell lines and xenografts would be useful for predicting the effectiveness and overcoming the resistance mechanisms of precision-based treatments.

## Materials and methods

### Pathological analysis

Informed consents for collection of samples during surgery and autopsy and their subsequent use for genetic analysis and other research purposes were obtained from the patient’s family.

The surgical and autopsy specimens were fixed with 20% buffered formalin and embedded in paraffin. Histopathological examination was performed on 4-μm-thick sections stained with hematoxylin and eosin (HE), and the Klüver-Barrera method. The pathological diagnosis was made on the basis of the WHO classification of tumors of the central nervous system (CNS) by an experienced pathologist (AK). Immunohistochemistry (IHC) was performed as described previously using primary antibodies against Ki-67 (1:100, monoclonal, clone MIB-1, DAKO, Glostrup, Denmark), BRAF V600E (1:50, monoclonal, clone VE1, Spring Bioscience, Pleasanton, CA, USA) [18, 19] and phosphorylated ERK (pERK: 1:200, monoclonal, 9101, Cell Signaling Technology (CST), Danvers, MA, USA).

Surgically obtained brain tissue and orthotopic brain tumor tissue were also subjected to electron microscopy. Glutaraldehyde-fixed small tissue blocks were post-fixed with 1% osmium tetroxide, dehydrated through a graded ethanol series, and embedded in Epon 812. Ultrathin sections were then cut and stained with uranyl acetate and lead citrate, and examined with a Hitachi H-7100 electron microscope at 75 kV.

### Establishment of a *BRAF* V600E-mutant epithelioid glioblastoma cell line

**Fig. 1:**
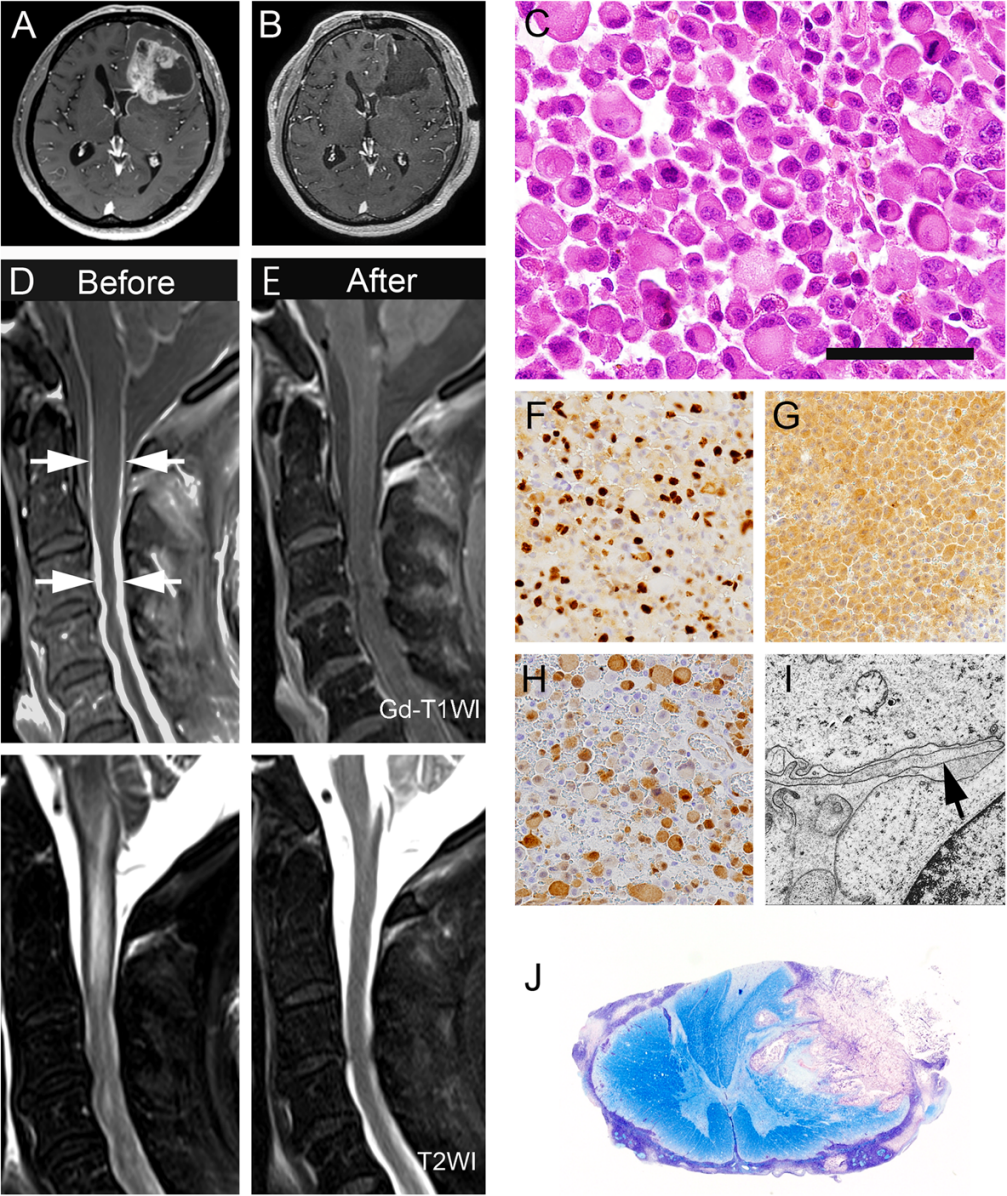
Clinicopathologic features of the patient. Preoperative (**a**) and postoperative (**b**) brain MR images of the lesion. Spinal cord MR images before (**d**) and after (**e**) combination therapy with BRAF and MEK inhibitor. Histopathologic features of the surgical (**c**, **f**, **g**, **h**, **i**; frontal lobe) and autopsy (**j**; second segment of the cervical cord) samples. T1-weighted gadolinium-enhanced image demonstrating enhancement of the well circumscribed tumor in the left frontal lobe (**a**) and confirming the subtotal resection except for enhancement of the lateral ventricle (**b**). Before targeted treatment, thick leptomeningeal dissemination (arrow) and syringomyelia are evident in the upper cervical cord on post-contrast MR images and T2-weighted images (**d**). After 4 weeks of treatment, T1-weighted gadolinium-enhanced images and T2-weighted images (**e**) reveal a dramatical radiological response to the therapy. The tumor cells are composed of discohesive, rounded cells with rhabdoid morphology, showing brisk mitotic activity (**c**). MIB-1 labeling index 36% (**f**). Positive immunoreactivity for BRAF V600E (**g**) and nuclear positivity for pERK (**h**). Ultrastructure of the tumor cells. A few foot processes (arrow) are not interwoven (**i**). Spinal cord invasion by the tumor cells with leptomeningeal dissemination (**j**). Scale bars **c**: 20 μm; **f**-**h**: 150 μm, **i**: 2 μm, **j**: 500 μm

The NGT41 cell line was established from a disseminated lesion of the cervical spinal cord at autopsy (Fig. 1j), in accord with the protocol approved by the institutional review board at Niigata University (#2016–2583). The specimen was minced with a scalpel and incubated in papain solution (Worthington Biochemical Corporation, Lakewood, NJ, USA) at 37 °C for 30 min with shaking to dissociate the tissue. The tissue was then triturated using a sterile pipette. After centrifugation of the suspension, the cell pellets were maintained in Dulbecco’s modified Eagle medium (DMEM) (Thermo Fisher Scientific, Waltham, MA, USA) supplemented with 10% fetal bovine serum (FBS) (Sigma Aldrich, St. Louis, MO, USA), 1% Antibiotic-Antimycotic (Thermo Fisher Scientific) and 5 μg/ml Plasmocin (InvivoGen, Toulouse, France).

### Cell lines and drugs

*BRAF* V600E-mutant glioblastoma cell lines, AM38 and DBTRG-05MG, were purchased from the Japanese Collection of Research Bioresources (JCRB) and the American Type Culture Collection (ATCC), respectively. Control *BRAF*-wildtype glioma cell lines, U87MG and T98G, were purchased from ATCC. All cell lines were grown in a humidified incubator at 37 °C under 5% CO_2_. NGT41, AM38, U87MG, and T98G cells were grown as adherent monolayer cultures in 10% FBS DMEM, and DBTRG-05MG cells were grown in 10% FBS RPMI1640 (Miltenyi Biotec, Aucurn, CA, USA) with Glutamax (Gibco, Paisley, UK). Dabrafenib (GSK2118436) was purchased from Selleck (Houston, TX, USA), and trametinib (GSK1120212) from AdooQ Bioscience (Irvine, CA, USA).

### Genetic characterization of tumor samples, cell lines and xenografts

The CANCERPLEX cancer genome panel (KEW Inc., Cambridge, MA, USA) of 435 cancer-related genes (Additional file 1: Table S1) was used to assess the tumor sample, as described previously [20]. DNA was extracted from fresh frozen tissue using the QIAamp Blood & Tissue Kit (Qiagen, Valencia, CA, USA) and from FFPE tumor tissue using the QIAmap DNA FFPE Tissue Kit (Qiagen). Direct sequencing of *BRAF* V600E and the *TERT* promoter was performed as reported previously [21]. Twenty nanograms of complimentary DNA was amplified using primers for the detection of *BRAF* V600E and the *TERT* promoter. The primer sequences were: *BRAF* V600E; forward 5′-TCATAATGCTTGCTCTGATAGGA-3′ and reverse 5′-GGCCAAAAATTTAATCAGTGGA-3′, TERT promoter; forward 5′-GTCCTGCCCCTTCACCTT-3′ and reverse 5′-CAGCGCTGCCTGAAACTC-3′. The PCR products were then sequenced on a 3130xl Genetic Analyzer (Applied Biosystems, Foster City, CA, USA) with a Big Dye Terminator v1.1 Cycle Sequencing Kit (Applied Biosystems) in accordance with the manufacturer’s instructions. Multiplex ligation-dependent probe amplification (MLPA) was selected to assess the *CDKN2A/2B* copy number. The SALSA MLPA probe mix P088-C2 (MARC-Holland, Amsterdam, the Netherlands) was used. In accordance with the manufacturer’s instructions, 250 ng of the DNA was denatured, hybridized, ligated, and subjected to PCR. The carboxyfluorescein (FAM)-labeled MLPA PCR products were separated by capillary electrophoresis on an ABI 3730xl DNA Analyzer (Applied Biosystems). The fragment lengths of the data were analyzed using Peak Scanner v2.0 (Thermo Fisher Scientific). Probe ratios below 0.4 were regarded as homozygous, and those from 0.4 to 0.7 were regarded as heterozygous deletions [22].

Droplet digital polymerase chain reaction (ddPCR) reagents and Primer/probe mix for *BRAF* V600E were purchased from Bio-Rad (Hercules, CA, USA). A 20-μL aliquot of PCR mix, composed of 10 μL of 2× ddPCR Supermix for Probes (No dUTP) (Bio-Rad), 1 μL ddPCR Mutation Assay (Bio-Rad), and 9 μL tumor DNA was loaded into each sample well of an 8-channel disposable droplet generator cartridge (Bio-Rad). An additional 70 μL of droplet generation oil (Bio-Rad) was loaded into the oil well for each channel. After droplet generation, droplets were transferred to a 96-well PCR plate and subjected to thermal cycling. Amplification of the 20 μL reaction mixture was carried out on a QX200 Droplet digital PCR system (Bio-Rad). After PCR, the 96-well PCR plate was transferred to a QX-200 droplet reader (Bio-Rad), and the data were analyzed using QuantaSoft analysis software (Bio-Rad). *BRAF* V600E mutation-specific signals were generated in the FAM channel, whereas *BRAF* V600E wildtype signals were generated in the HEX channel.

### Cell viability

Fifteen hundred cells per 100 μL medium were seeded into 96-well plates for one day and 10 μL of each drug was applied for 72 h. As a background control (0% control), 0.2% Triton X-100 was added to the control wells. To each well was then added 10 μL of WST-8 (Nakalai Tesque, Kyoto, Japan), and the cells were incubated for 4 h. Absorbance at 450 nm was measured in each well using ELx808 (BioTek Instruments, Winooski, VT, USA). Cell viability was quantified as: cell viability (%) = (Asample - a0%) / (a100% - a0%) × 100, where Asample is the sample absorbance, a0% is the average 0% control absorbance, and a100% is the average 100% control absorbance.

The normalized growth rate inhibition (GR) analysis was performed to minimize cell line-dependent differences in rate of cell division [23]. Fifteen hundred cells per 100 μL medium were seeded into 96-well plates for one day and cell viability was measured at the time of treatment, and at 48 and 72 h after each drug administration using WST-8. Dabrafenib was diluted ranging from 1 nM to 1000 nM and trametinib from 0.1 nM to 100 nM in 7 increments. 0.5% DMSO was added to the control well. GR metrics and the GR_50_ was calculated using the Online GR Calculator (www.grcalculator.org/grcalculator).

### Apoptosis assay and cell cycle analysis

Apoptosis and the cell cycle were assessed using a Muse™ Cell Analyzer (Millipore, Billerica, MA, USA) with Annexin V and Dead Cell Assay and Cell Cycle analysis kits following the manufacturer’s instructions, as described previously [24]. In brief, after the indicated treatments (dabrafenib; 10 nM, trametinib; 1 nM, combined; dabrafenib 10 nM + trametinib 1 nM) for 48 h, the cells were harvested, centrifuged at 300×g for 5 min, and washed once with 1 × PBS. For the apoptosis assays, a 100-μL cell suspension was labeled with Annexin V and Dead Cell Reagent (7-Amino-Actinomicyn D (7-AAD)) for 20 min at room temperature in the dark, and then analyzed. For cell cycle analysis, the cells were fixed in 70% ethanol for at least 3 h, then washed with PBS and stained with 200 μL propidium iodide (PI) for 30 min. After staining, the cells were processed for cell cycle analysis.

### Western blotting

Proteins were extracted using cell lysis buffer, and Western blotting was performed as described previously [25]. The blotted membranes were probed with anti-MEK (1:500, CST), anti-pMEK (1:500, CST), anti-ERK (1:1000, CST), anti-pERK (1:500, CST) and anti-β-actin (1:000, CST), and the signals were detected with an ECL system (Bio-Rad, Hercules, CA, USA).

### Subcutaneous and intracranial xenografts

Four-to-five-week-old male BALB-c nu/nu mice were purchased from Charles River Laboratories Inc. (Yokohama, Japan) and housed under aseptic conditions, which included filtered air and sterilized food, water, bedding, and cages. The mice were anesthetized with medetomidine hydrochloride (0.3 mg/kg), midazolam (4.0 mg/kg) and butorphanol tartrate (5.0 mg/kg) by intraperitoneal injection.

For the subcutaneous tumor model, 1 × 10^6^ NGT41 cells were suspended in 50 μL of Neurobasal Medium (Gibco) and injected subcutaneously into the hip. To investigate the efficacy of treatment, these mice received vehicle control (0.5% hydroxypropyl methylcellulose plus 0.2% Tween 80, and 20% dimethyl-sulfoxide (DMSO)) or combined treatment (20 mg/kg dabrafenib and 2 mg/kg trametinib solubilized in 20% DMSO) by oral gavage for 10 days. The tumors were measured daily with calipers, and tumor volume was calculated using the formula: Tumor volume (mm^3^) = Length (mm) × Width (mm)^2^/2 [26]. Two additional mice from each treatment group were used for histopathological and Western blot analyses as described above. These mice were sacrificed at 2 h after completion of treatment on the fifth day in order to collect tumor tissues.

For the intracranial xenograft model, a NGT41 cell suspension (1 × 10^5^ cells in 2 μL of Neurobasal Medium) was stereotactically injected into the right caudate putamen at 1.0 mm to the right of the midline, just behind the bregma, and at 3.0 mm depth. The mice were monitored daily and euthanized if body weight loss exceeded 20%, or if they developed neurological symptoms indicative of tumor burden. Brain tissue was subsequently resected and subjected to formalin fixation and paraffin embedding.

All animal studies were approved by the Animal Use and Care Committee of Niigata University, and all of the animals were cared for and treated humanely in accordance with the Institutional Guidelines for Experiments Using Animals.

### Statistical analysis

Differences between three or more groups were assessed using two-way analysis of variance (ANOVA) with the *p*-value threshold adjusted by Bonferroni’s correction (i.e. *p* < 0.025 for 3 groups, *p* < 0.0125 for 4 groups) unless otherwise specified. Error bars represent standard error of the mean. Kaplan-Meier survival curves for intracranial xenografts were assessed by Log-rank test, and *p* < 0.05 was considered statistically significant unless otherwise specified. All statistical tests were performed using the GraphPad Prism 6 software package (GraphPad Software, La Jolla, CA, USA).

## Results

### Case report

A 57-year-old man presented with headaches and slight dysphasia. Post-contrast MR images revealed a well circumscribed mass lesion measuring 6 cm × 5 cm at the left frontal lobe, adjacent to the lateral ventricle. Subependymal enhancement was noted inside the left lateral ventricle (Fig. 1a). The intraparenchymal enhancing tumor was subtotally removed; the portion of the mass surrounding the lateral ventricle was left in situ (Fig. 1b). Examination of HE sections showed highly cellular, discohesive and medium-sized rounded cells with abundant eosinophilic cytoplasm and laterally positioned nuclei (Fig. 1c). Microvascular proliferation was rare; however, prominent necrosis was observed. The MIB-1 labeling index was about 36% (Fig. 1f). Immunohistochemistry showed that the tumor cells were positive for BRAF V600E (Fig. 1g) and pERK (Fig. 1h). Ultrastructurally, the foot processes were not interwoven and lacked the macula adherens, unlike the common appearance in glioblastoma (Fig. 1i). The pathological diagnosis was epithelioid GBM. After surgery, the patient received intensity-modulated radiation therapy and concomitant temozolomide. During radiation, the patient became comatose and post-contrast brain MR images revealed dissemination and hydrocephalus. External ventricular drainage was performed to control the hydrocephalus and irradiation was continued, enlarging the radiation field to include the whole brain. After completion of concomitant treatment, a ventriculo-peritoneal shunt was performed, but the patient subsequently developed paraplegia. Spinal MR images revealed thick spinal dissemination and syringomyelia (Fig. 1d).

*BRAF* V600E and *TERT* promoter (C250T) mutations, and *CDKN2A/2B* loss were identified using the CANCERPLEX comprehensive genomic panel. No other genetic mutations were found. These genetic changes were confirmed by Sanger sequencing and MLPA analysis (Additional file 2: Figure S1). The fractional abundance (FA) of *BRAF* V600E calculated by ddPCR was 52.1% (Additional file 2: Figure S2). Although whole spinal irradiation was commenced, the patient’s neurological conditions clearly worsened and a decline in the level of consciousness and dysphagia were observed, thus irradiation was stopped at 15 Gy (5 fractions). Following accelerated IRB approval for off-label use and written informed consent, the patient received 150 mg of dabrafenib twice a day and 2 mg of trametinib daily. A total of 4 weeks of targeted treatment was administered, alternating with 5 more fractions of whole spinal radiation to a total dose of 30 Gy. After 4 weeks of treatment, spinal MR images demonstrated almost complete disappearance of the dissemination and syringomyelia (Fig. 1e). Unfortunately, the patient’s paraplegia did not improve and he was taken off targeted treatment, mainly due to lack of funding. The patient was subsequently treated with temozolomide and bevacizumab, but succumbed to the disease 8 months after surgery.

At autopsy, macroscopic examination of the spinal cord revealed a thickened and whitish subarachnoid membrane, and the subarachnoid space was largely filled with tumor cells showing invasion into the dorsal part of the second segment of the cervical cord (Fig. 1j). A few remaining tumor cells formed small perivascular sheets within and around the surgically treated lesion in the left frontal lobe. However, no tumor cells were detectable in other parenchymal areas and visceral organs. Morphologically, the tumor cells shared features similar to those in the surgical sample, except for the mucinous component at autopsy.

### Characterization of NGT41 cells and xenografts

NGT41 cells displayed robust growth and sustainable propagation in adherent cell culture. The NGT41 cell line retained the *BRAF* V600E and *TERT* promoter (C250T) mutation, and heterozygous deletion of *CDKN2A/2B* (probe ratios were between 0.4 and 0.7), observed in the tissues obtained at surgery and autopsy (Additional file 2: Figure S1A, B).

**Fig. 2:**
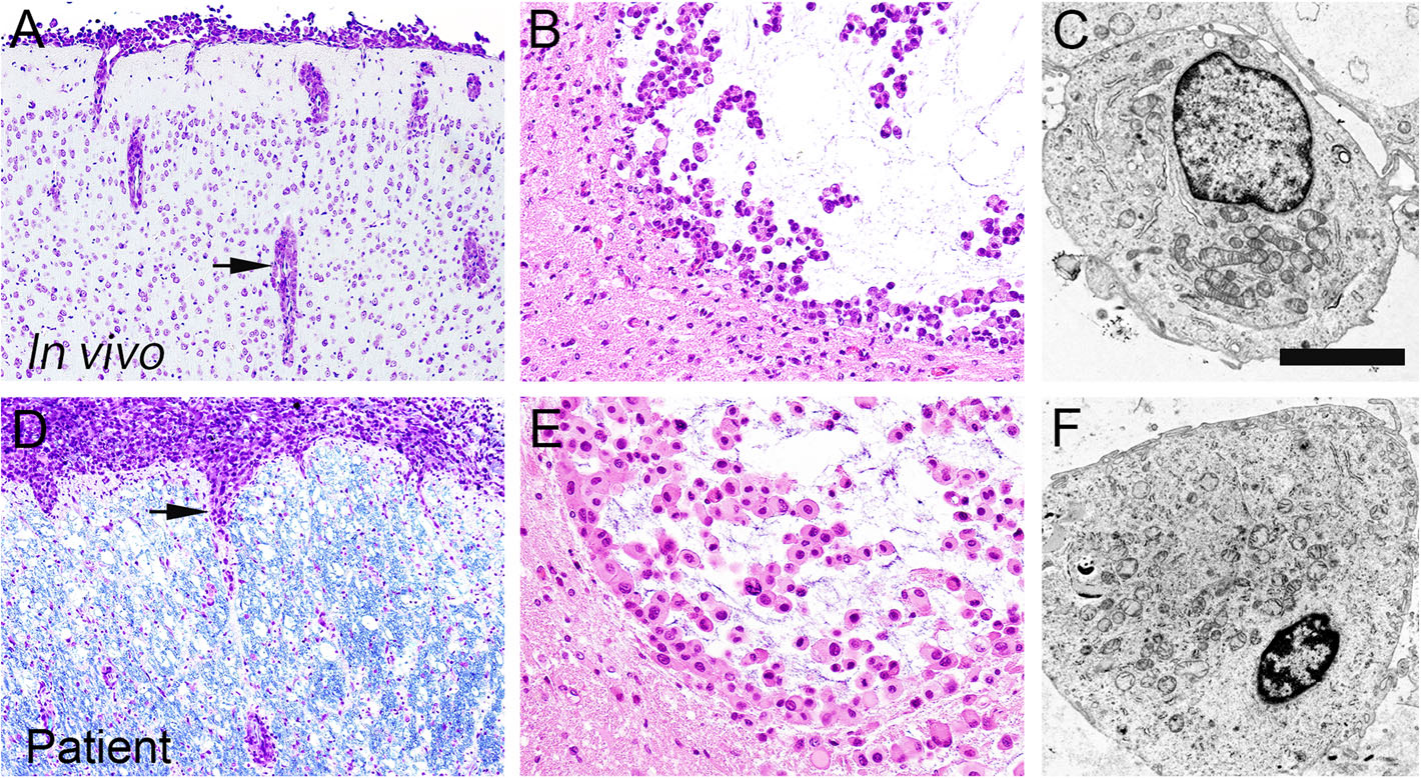
Morphological similarity of the tumor cells between those in the orthotopic xenograft (**a**, **b**, **c**) and those from the patient (**d**, **e**, **f**). The orthotopic xenografts share strikingly similar characteristics to those of the autopsy specimen, including the mode of tumor infiltration (**a**, **d**) and the light microscopic (**b**, **e**) and ultrastructural (**c**, **f**) morphology of the tumor cells. Scale bars **a**, **d**: 200 μm; **b**, **e**: 50 μm; **c**, F: 2 μm

The intracranial xenograft tumors formed well circumscribed masses in the brain, and showed dissemination into the subarachnoid with perivascular infiltration (Fig. 2a), morphologically resembling epithelioid cells with a mucinous component (Fig. 2b). These features closely resembled the patient’s tumor at autopsy (Fig. 2d, e). Moreover, ultrastructural examination of the intracranial xenograft tumors revealed rounded tumor cells characterized by few foot processes and rich cytoplasmic organelles (Fig. 2c), similar to the tissue obtained at surgery (Fig. 2f).

### In vitro studies

**Fig. 3:**
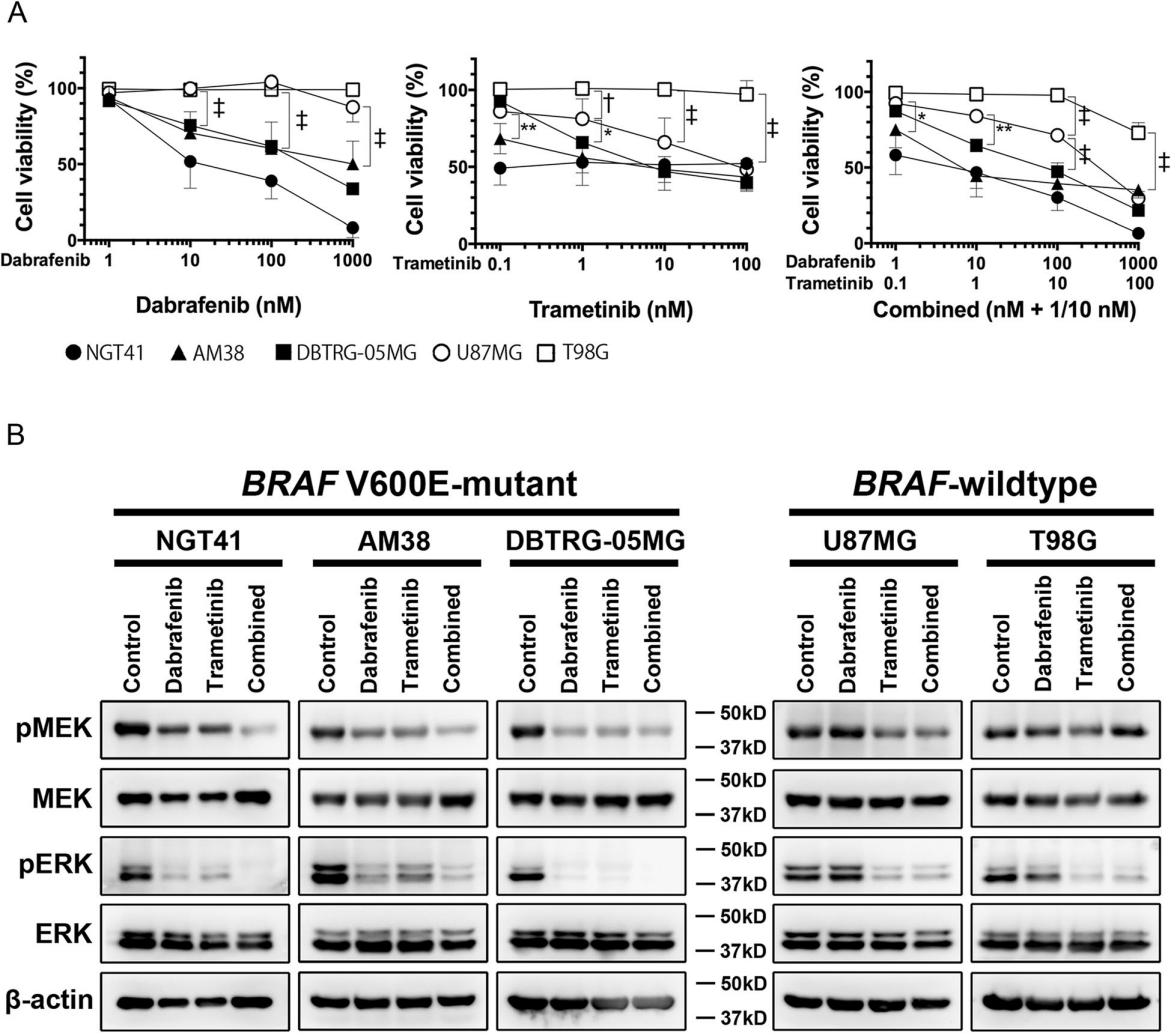
Effectiveness of dabrafenib and trametinib against *BRAF* V600E-mutant GBM cell lines in vitro*.* Targeted treatment reduced the viability of NGT41 and other *BRAF* V600E-mutant glioma cell lines (AM38, DBTRG-05MG) relative to *BRAF-*wildtype glioma cell lines (U87MG, T98G). (*n* ≧6, ^*^p < 0.05, ^**^p < 0.01, ^†^*p* < 0.001, ^‡^*p* < 0.0001; Two-way ANOVA) (**a**). Marked inhibition of phosphorylated MEK and ERK was observed in *BRAF* V600E-mutant cell lines. The expression of phosphorylated ERK was suppressed by trametinib treatment, regardless of *BRAF* status (**b**)

We evaluated the sensitivity of NGT41 and other *BRAF* V600E-mutant cell lines to treatment with dabrafenib and/or trametinib. The cell lines were treated with either dabrafenib (at 0, 1, 10, 100, or 1000 nM) or trametinib (at 0, 0.1, 1, 10, or 100 nM), or with both (combined; dabrafenib + 1/10 trametinib) for 72 h. All *BRAF* V600E-mutant cell lines were sensitive to both drugs. In NGT41, addition of trametinib did not significantly reduce cell viability compared to dabrafenib alone. Among *BRAF* V600E wildtype cell lines, U87MG showed a response to trametinib, consistent with a previous report (Fig. 3a) [27]. Next, we calculated growth rate inhibition in NGT41 and U87MG to account for possible cell line-dependent differences in rate of cell division. Indeed, combination treatment in NGT41 showed a dramatic reduction in growth rate, whereas only a minimal reduction in growth rate was observed after combined treatment in U87MG (Additional file 2: Figure S3).

To assess the effects of dabrafenib and trametinib on the MAPK signaling pathway, we used Western blot analysis to examine the protein expression of key pathway components. Cells were treated with the blank control (0.5% DMSO) or each drug (10 nM dabrafenib or 1 nM trametinib or combined; 10 nM dabrafenib + 1 nM trametinib) for 1 h. Treatment with dabrafenib reduced the expression of both pMEK and pERK in *BRAF* V600E-mutant cell lines. Treatment with trametinib reduced the expression of pERK regardless of *BRAF* V600E status (Fig. 3b). In NGT41, combined treatment led to profound decreases in pMEK and pERK.

*BRAF* V600E-mutant cell lines, but not *BRAF* wildtype cell lines, treated with dabrafenib and trametinib in combination exhibited significantly greater apoptosis (Additional file 2: Figure S4A; one-way ANOVA with Bonferroni’s correction) and G0/G1 arrest (Additional file 2: Figure S4B).

### In vivo studies

**Fig. 4:**
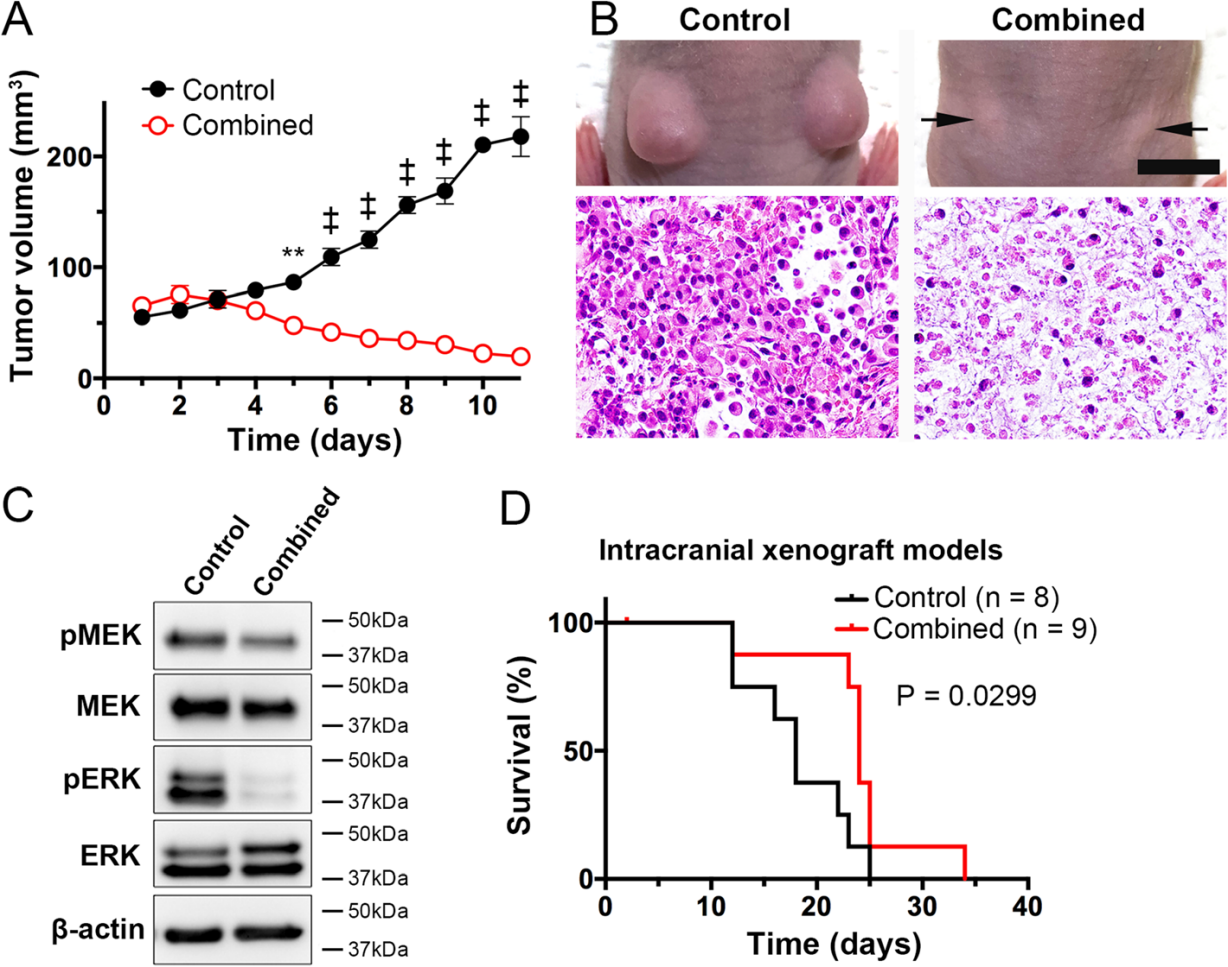
Efficacy of combined treatment against subcutaneous and intracranial xenograft models. In the combined treatment group, tumor volume was significantly suppressed relative to the control group. (*n* = 4, error bars represent ± SEM^, **^p = 0.0021, ^‡^*p* < 0.0001; Two-way ANOVA) (**a**). Representative macro and micro images of the tumors from mice implanted subcutaneously with NGT41 cells (**b**). Western blot analysis demonstrated that expression of phosphorylated ERK was drastically inhibited by the combined treatment (**c**). Combined dabrafenib and trametinib treatment significantly prolonged the survival of mice in the intracranial model relative to the control (*p* = 0.0299, Log Rank test) (**d**). Scale bars **a**: 10 mm; **b**: 50 μm

As the combination of BRAF and MEK inhibitors showed increased efficacy against NGT41 in vitro, we next tested the antitumor activity of this combination therapy using orthotopic human tumor xenografts. We transplanted NGT41 subcutaneously into the hip of BALB-c nu/nu mice, and treated the mice with control vehicle or dabrafenib and trametinib in combination daily by oral administration from day 5 of implantation for 10 days. Combined treatment significantly suppressed tumor growth in vivo compared with the vehicle control (79.45 ± 8.27 (mean ± SEM) vs 61.24 ± 9.36 *p* < 0.0021 at day 5, and *p* < 0.0001 at days 6–11 after initiation of treatment) (Fig. 4a). We removed the tumors from the mice after completion of treatment on day 5. Histopathological examination showed that combined treatment markedly decreased the numbers of viable tumor cells (Fig. 4b). Western blot analysis showed that combined treatment suppressed the expression of pMEK and pERK in vivo (Fig. 4c).

We then sought to prove the efficacy of combination treatment on intracranial tumor models. We transplanted NGT41 cells into the mouse brain by stereotactic injection and treated the mice with vehicle control or combined treatment for 14 days. Combination treatment resulted in only a modest improvement in survival (*p* = 0.0299, Log Rank test) (Fig. 4d). Western blot analysis of the tumor in an intracranial xenograft mouse treated with the combined treatment for 3 days before sacrificing showed marked reduction of pMEK and pERK after treatment compared to control (Additional file 2: Figure S5A). Also, serial body weight measurements were unchanged in the combination treatment group compared to control (Additional file 2: Figure S5B). HE sections of brain tumors in the treatment group at endpoint showed tumors of the same high-cellularity as the control group (Additional file 2: Figure S5C).

## Discussion

Recent phase 3 clinical trials have advocated the usefulness of a combination of the BRAF inhibitor dabrafenib and the MEK inhibitor trametinib as first-line treatment for *BRAF* V600E-mutant metastatic melanomas [28, 29], as BRAF inhibitors alone cause resistance by reactivation of the MAPK pathway due to PDGFRB upregulation or *NRAS* mutation [30]. A preclinical study has demonstrated the efficacy of a combination of the BRAF inhibitor PLX4720 and the MEK inhibitor PD0325901 against *BRAF* V600E-mutant GBM [31]. The present in vitro and in vivo results were generally in agreement with those previous data.

The VE-BASKET study showed that vemurafenib alone was effective in only 9.1% of malignant diffuse gliomas, compared to 42.9% of PXAs [17]. This suggests that in malignant gliomas harboring *BRAF* V600E mutations, other pathways such as PI3K-Akt-mTOR pathway are also activated, *BRAF* V600E mutations are merely one of multiple driving mutations and may be present in a proportion of tumor cells, whereas in epithelioid GBMs, *BRAF* V600E mutation is likely a driving and recurrent event [7]. In the present case, FA of *BRAF* V600E by ddPCR was high (Additional file 2: Figure S2).

Interestingly, a recent series of 15 anaplastic PXAs reported that 100% had RAF alterations (mostly *BRAF* V600E and occasional *BRAF* or *RAF1* fusions), 100% had biallelic inactivation of *CDKN2A*, and 47% had *TERT* promoter mutations [32], revealing a striking similarity of genetic alterations between anaplastic PXAs and epithelioid GBMs. Furthermore, a glioma mouse model obtained by transferring the activated forms of BRAF V600E, KRAS and AKT to neural progenitor cells in *Ink4a/Arf* ^*lox/lox*^ mice has been reported [33]. Deletion of *Ink4a*, an important tumor suppressor, produces the same effect as *CDKN2A* loss (which encodes p16INK4a) in gliomas. Induction of *BRAF* V600E alone was not tumorigenic; *Ink4a/Arf* loss in combination produced well-demarcated gliomas showing evidence of growth into the subarachnoid space, recapitulating the characteristics of epithelioid GBM. Thus, the epithelioid GBM model with *BRAF* V600E mutation in combination with *CDKN2A* loss we established recapitulates the characteristics of previously published models.

Analysis of The Cancer Genome Atlas (TCGA) database included in R2: Genomics Analysis and Visualization Platform (http://r2.amc.nl) showed that *BRAF* mutations were significantly correlated with *CDKN2A* alterations (*p* = 0.025) (Additional file 2: Figure S6A) and *TERT* promoter mutations (*p* = 7.03e-03) (Additional file 2: Figure S6B). These three confirmed genetic alterations may be closely related to tumorigenesis in epithelioid GBM.

A recent report based on integrated molecular analysis has proposed that epithelioid GBM should be stratified into three subsets: a PXA subset with a high percentage of *BRAF* V600E mutations but a relatively low percentage of *TERT* promoter mutations, an adult *IDH*-wildtype GBM subset with a relatively low percentage of *BRAF* V600E mutations but a high percentage of *TERT* promoter mutations, and a pediatric RTK1 subset not harboring either mutation [34]. This previous report illustrates the molecular and pathological complexity of epithelioid GBM, suggesting the need for cancer panel assessment before precision medicine can be considered.

To our knowledge, this is one of the first reports of epithelioid GBM characterized by leptomeningeal dissemination showing a dramatic response to combined BRAF and MEK inhibitors. Epithelioid GBMs with *BRAF* V600E mutation showing leptomeningeal dissemination have an especially poor prognosis with a patient survival of 1–3 months [1–3], whereas the present patient survived for 8 months after only 4 weeks of targeted therapy. Other previous reports have indicated sensitivity to BRAF inhibitor alone for gliomas with *BRAF* V600E mutations, the majority being gangliogliomas in children and PXAs in adults [35]. More recent reports have documented successful treatment of high-grade gliomas with *BRAF* V600E mutations using a combination of dabrafenib and trametinib [35–37]. A case report illustrates stable disease for 10 months in a *BRAF*-mutant epithelioid GBM patient treated with dabrafenib alone [38]; a very recently published report shows dramatic, albeit transient response to dabrafenib and trametinib in two *BRAF* V600E-mutant epithelioid glioblastomas [39]. In the present case, the patient’s paraplegia did not improve despite dramatic radiographical response. Findings at autopsy suggested that the tumor cells had already invaded into the spinal cord, causing irreversible destructive changes before initiation of targeted therapy.

We developed the NGT41 cell line using autopsied tumor tissue, and established a xenograft showing characteristics closely resembling those of the autopsied tumor itself, with invasion into the subarachnoid and perivascular spaces, and a mucinous stroma (Fig. 2a, b). Treatment experiments were conducted both in vitro and in vivo, and while we were able to demonstrate efficacy of the combination therapy, some of the results were not as robust as we had expected. One explanation for this is that the cell line we established was not treatment-naive. For instance, mucin was seen in the xenograft and was present in the medium after culture, whereas it was not observed at all in the initial tumor. Furthermore, it is known that additional genetic changes can occur in long-term-cultured cell lines [40]. Further genetic and epigenetic evaluation of the initial tumor, autopsy specimen, and cell line/ xenograft should be carried out in order to assess any treatment-related changes. In the patient, spinal irradiation was performed in conjunction with dabrafenib and trametinib, possibly enhancing the cytotoxicity of treatment [41], but clinical response was observed soon after starting the combined BRAF inhibitor and MEK inhibitor treatment, suggesting that targeted treatment was the main reason for the dramatic effect. Results of pMEK and pERK analysis in the intracranial tumor revealed that drug properly entered the brain (Additional file 2: Figure S5A). Also, adverse effects of the drugs were tolerable (Additional file 2: Figure S5B). Morphological findings of the HE sections suggested that tumors in the treatment group rapidly recurred after planned cessation of treatment at 14 days (Additional file 2: Figure S5C), supporting the notion that dabrafenib and trametinib should be continued until relapse. Despite these limitations, patient-derived cell lines may predict the effectiveness of targeted therapy and lead to an improved understanding of the tumor biology. Furthermore, establishment of this cell line and xenograft will potentially lead to the understanding of resistance mechanisms to BRAF and MEK inhibition and development of strategies to circumvent this resistance.

## Conclusions

Dabrafenib and trametinib in combination with spinal radiation elicited a dramatic response in a patient with epithelioid GBM harboring *BRAF* V600E mutation, and characterized by spinal dissemination. Moreover, we established a cell line retaining the *BRAF* V600E mutation, which morphologically resembled the epithelioid glioblastoma, and were able to evaluate the efficacy of combined treatment. Further research using this cell line is expected to help development of new strategies to mitigate the development of resistance and augment the response to BRAF and MEK inhibition.

## Additional files

Additional file 1: **Table S1.** A table listing the 435 genes in the comprehensive genomic sequencing panel. (TIF 7228 kb)

Additional file 2: **Figure S1.** Genetic profiles of surgical tissue and the NGT41 cell line. *BRAF* V600E and *TERT* promotor (C250T) mutation was confirmed by Sanger sequencing **(A)**, and heterozygous loss of *CDKN2A/2B* was identified by the multiplex ligation-dependent probe amplification method **(B)**. **Figure S2.** Evaluation of *BRAF* V600E using ddPCR. Tumor DNA was extracted from the area of vivid tumor cells in the FFPE tissue by laser microdissection **(A)**. Fractional abundance (FA) of mutated *BRAF* V600E was calculated as copies of mutated DNA/(copies of mutated DNA + wildtype DNA) **(B)**. Scale bar A: 200 μm. **Figure S3.** Calculation of growth rate value in NGT41 and U87MG after combination treatment. Dose response curves on relative cell count showed marked response to BRAF and/or MEK inhibitor treatment in NGT41 (A), but minimal reduction in U87MG (B). **Figure S4.** BRAF and MEK inhibitor induced greater apoptosis and G0/G1 arrest in the NGT41 cell line. In *BRAF* V600E-mutant cell lines, each treatment significantly increased the number of apoptotic cells (*n* = 3, ^*^p < 0.05, ^**^p < 0.01; Two-way ANOVA) **(A)**. G0/G1 arrest was induced by each treatment in *BRAF* V600E mutant-cell lines, whereas no response was observed in *BRAF*-wildtype cell lines (*n* = 3) **(B)**. **Figure S5.** Effect of BRAF and MEK inhibitor in the intracranial model. pMEK and pERK were markedly suppressed in the treatment group **(A)**. Serial body weight calculations in the treatment group were virtually the same as in the control group **(B)**. Histological appearance of intracranial tumor in the treatment group at endpoint was similar to that of the control group **(C)**. Scale bar C: 50 μm. **Figure S6.** Analysis of the TCGA database included in R2: Genomics Analysis and Visualization Platform showed that *BRAF* mutations were significantly correlated with *CDKN2A* alterations (*p* = 0.025) **(A)** and *TERT* promoter mutations (*p* = 7.03e-03) **(B)**. (ZIP 8066 kb)

## Data Availability

The datasets used during the current study are available from the corresponding author on reasonable request. The TCGA datasets generated and/or analyzed during the current study are available in the R2 analysis and visualization platform (http://r2.amc.nl).

## Abbreviations

7-AAD: 7-Amino-Actinomycin D
ANOVA: analysis of variance
ATCC: American Type Culture Collection
CNS: Central nervous system
ddPCR: droplet digital polymerase chain reaction
DMEM: Dulbecco’s modified Eagle medium
DMSO: Dimethyl-sulfoxide
FA: Fractional abundance
FAM: carboxyfluorescein
FBS: Fetal bovine serum
GBM: Glioblastoma
GR: Growth ratio
HE: Hematoxylin and eosin
IHC: Immunohistochemistry
IRB: Institutional review board
JCRB: Japanese Collection of Research Bioresources
MLPA: Multiplex ligation-dependent probe amplification
PI: Propidium iodide
PXA: Pleomorphic xanthoastrocytoma
TCGA: The Cancer Genome Atlas
