# Supplementary figures and images for: Dramatic response of BRAF V600E-mutant epithelioid glioblastoma to combination therapy with BRAF and MEK inhibitor: establishment and xenograft of a cell line to predict clinical efficacy
Source: Acta Neuropathol Commun. 2019 Jul 25;7:119. doi: 10.1186/s40478-019-0774-7 (PMC6659204; doi:10.1186/s40478-019-0774-7)

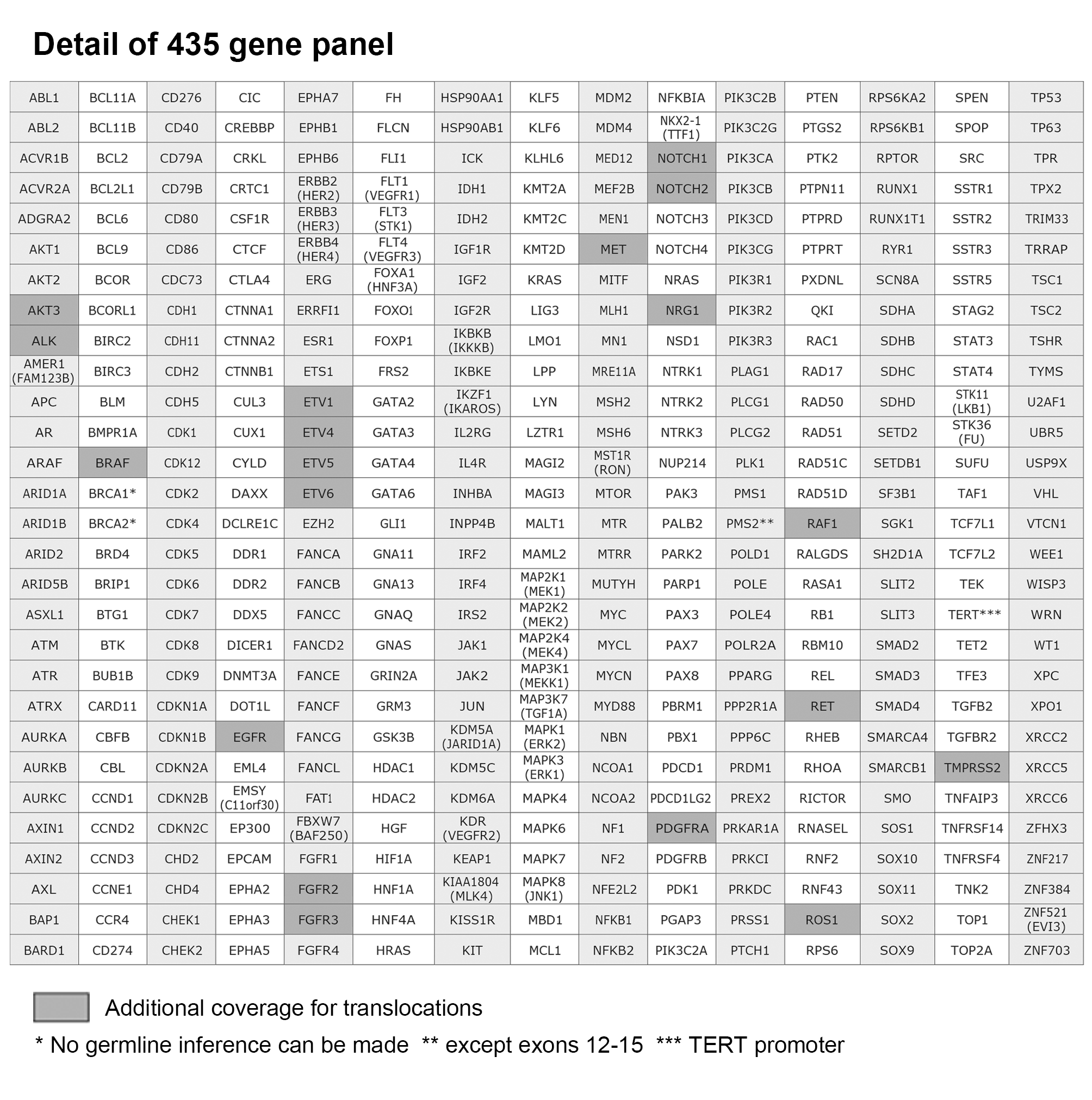

Supplement: Supplementary file 1 — Table S1. A table listing the 435 genes in the comprehensive genomic sequencing panel. (TIF 7228 kb) [file 40478_2019_774_MOESM1_ESM.tif]

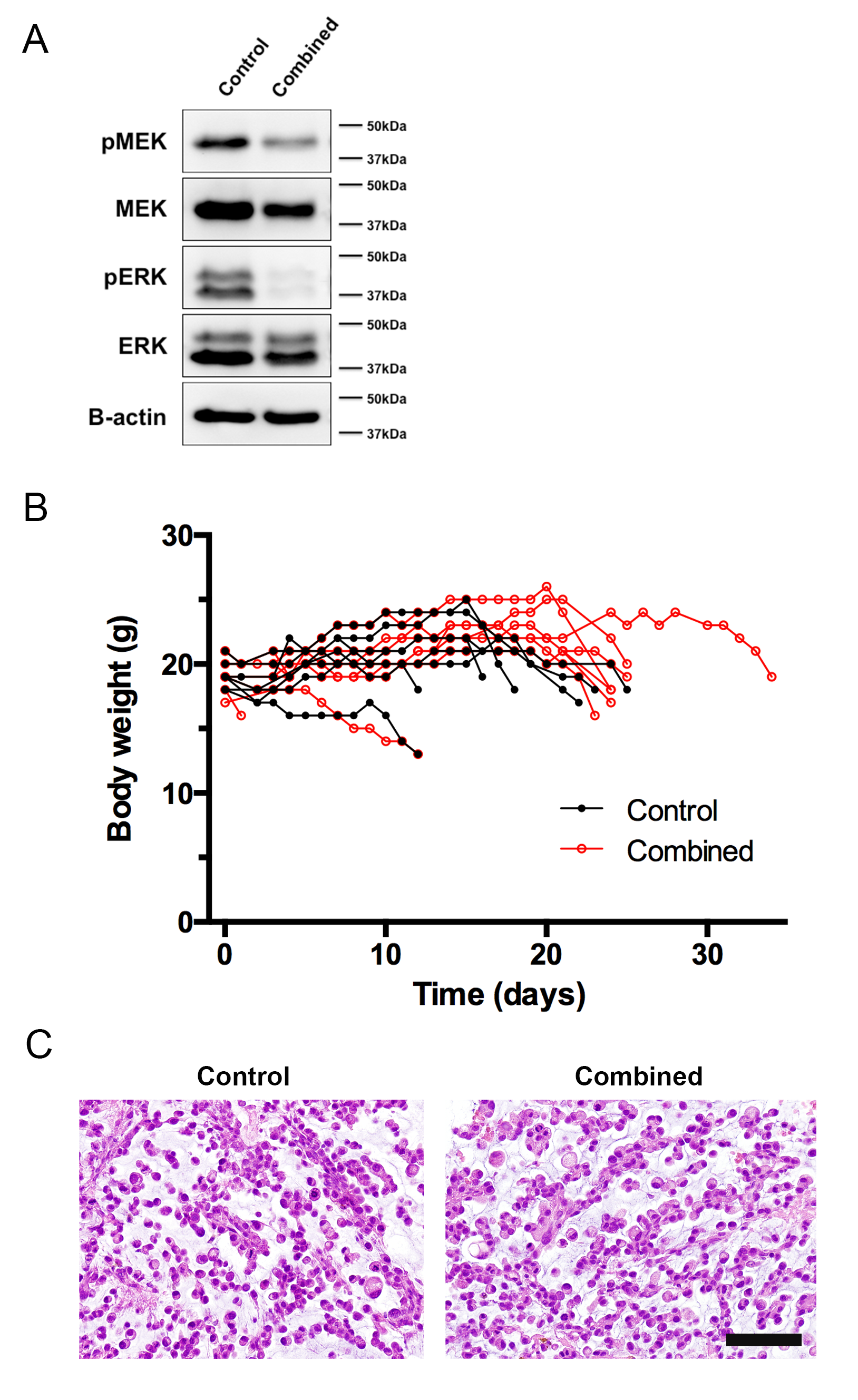

Supplement: Supplementary file 2 — Figure S1. Genetic profiles of surgical tissue and the NGT41 cell line. BRAF V600E and TERT promotor (C250T) mutation was confirmed by Sanger sequencing (A), and heterozygous loss of CDKN2A/2B was identified by the multiplex ligation-dependent probe amplification method (B). Figure S2. Evaluation of BRAF V600E using ddPCR. Tumor DNA was extracted from the area of vivid tumor cells in the FFPE tissue by laser microdissection (A). Fractional abundance (FA) of mutated BRAF V600E was calculated as copies of mutated DNA/(copies of mutated DNA + wildtype DNA) (B). Scale bar A: 200 μm. Figure S3. Calculation of growth rate value in NGT41 and U87MG after combination treatment. Dose response curves on relative cell count showed marked response to BRAF and/or MEK inhibitor treatment in NGT41 (A), but minimal reduction in U87MG (B). Figure S4. BRAF and MEK inhibitor induced greater apoptosis and G0/G1 arrest in the NGT41 cell line. In BRAF V600E-mutant cell lines, each treatment significantly increased the number of apoptotic cells (n = 3, *p < 0.05, **p < 0.01; Two-way ANOVA) (A). G0/G1 arrest was induced by each treatment in BRAF V600E mutant-cell lines, whereas no response was observed in BRAF-wildtype cell lines (n = 3) (B). Figure S5. Effect of BRAF and MEK inhibitor in the intracranial model. pMEK and pERK were markedly suppressed in the treatment group (A). Serial body weight calculations in the treatment group were virtually the same as in the control group (B). Histological appearance of intracranial tumor in the treatment group at endpoint was similar to that of the control group (C). Scale bar C: 50 μm. Figure S6. Analysis of the TCGA database included in R2: Genomics Analysis and Visualization Platform showed that BRAF mutations were significantly correlated with CDKN2A alterations (p = 0.025) (A) and TERT promoter mutations (p = 7.03e-03) (B). (ZIP 8066 kb) [file 40478_2019_774_MOESM2_ESM.zip › Additional file 2-Fig.S5.tif]

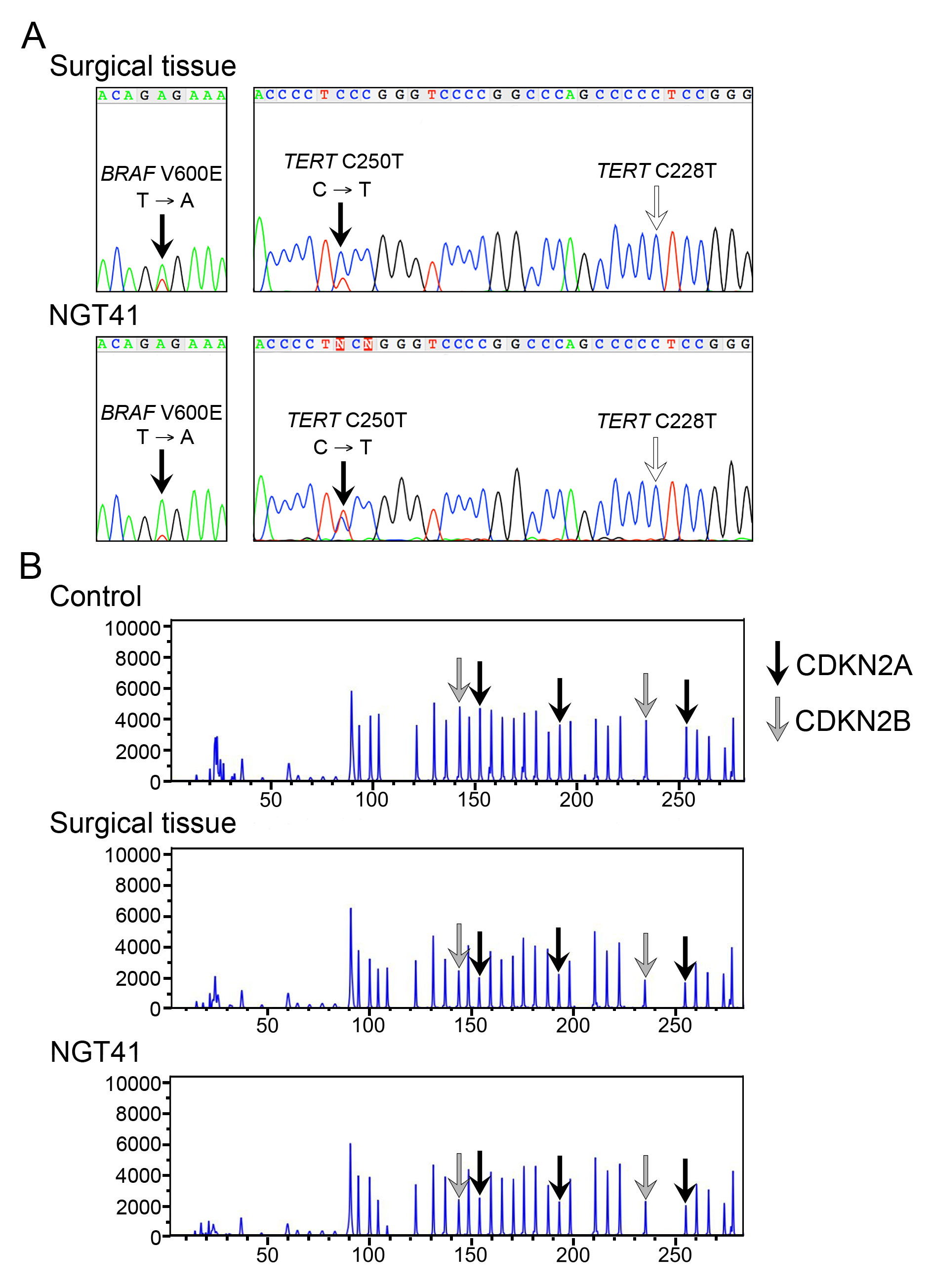

Supplement: Supplementary file 2 — Figure S1. Genetic profiles of surgical tissue and the NGT41 cell line. BRAF V600E and TERT promotor (C250T) mutation was confirmed by Sanger sequencing (A), and heterozygous loss of CDKN2A/2B was identified by the multiplex ligation-dependent probe amplification method (B). Figure S2. Evaluation of BRAF V600E using ddPCR. Tumor DNA was extracted from the area of vivid tumor cells in the FFPE tissue by laser microdissection (A). Fractional abundance (FA) of mutated BRAF V600E was calculated as copies of mutated DNA/(copies of mutated DNA + wildtype DNA) (B). Scale bar A: 200 μm. Figure S3. Calculation of growth rate value in NGT41 and U87MG after combination treatment. Dose response curves on relative cell count showed marked response to BRAF and/or MEK inhibitor treatment in NGT41 (A), but minimal reduction in U87MG (B). Figure S4. BRAF and MEK inhibitor induced greater apoptosis and G0/G1 arrest in the NGT41 cell line. In BRAF V600E-mutant cell lines, each treatment significantly increased the number of apoptotic cells (n = 3, *p < 0.05, **p < 0.01; Two-way ANOVA) (A). G0/G1 arrest was induced by each treatment in BRAF V600E mutant-cell lines, whereas no response was observed in BRAF-wildtype cell lines (n = 3) (B). Figure S5. Effect of BRAF and MEK inhibitor in the intracranial model. pMEK and pERK were markedly suppressed in the treatment group (A). Serial body weight calculations in the treatment group were virtually the same as in the control group (B). Histological appearance of intracranial tumor in the treatment group at endpoint was similar to that of the control group (C). Scale bar C: 50 μm. Figure S6. Analysis of the TCGA database included in R2: Genomics Analysis and Visualization Platform showed that BRAF mutations were significantly correlated with CDKN2A alterations (p = 0.025) (A) and TERT promoter mutations (p = 7.03e-03) (B). (ZIP 8066 kb) [file 40478_2019_774_MOESM2_ESM.zip › Additional file-2-Fig.S1.tif]

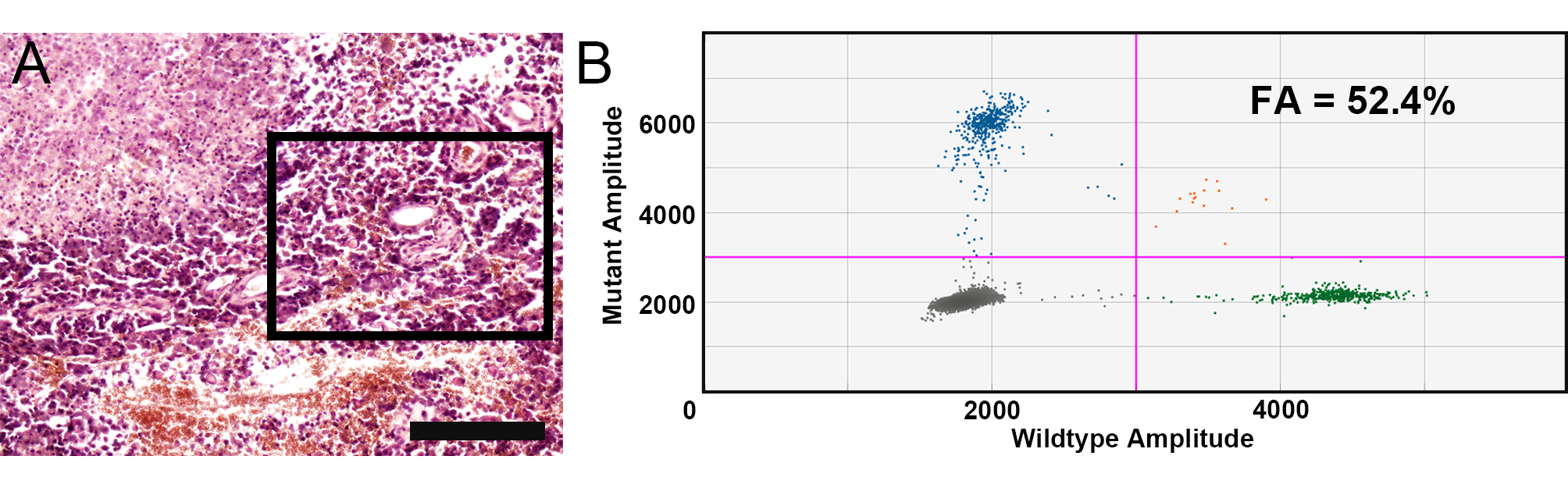

Supplement: Supplementary file 2 — Figure S1. Genetic profiles of surgical tissue and the NGT41 cell line. BRAF V600E and TERT promotor (C250T) mutation was confirmed by Sanger sequencing (A), and heterozygous loss of CDKN2A/2B was identified by the multiplex ligation-dependent probe amplification method (B). Figure S2. Evaluation of BRAF V600E using ddPCR. Tumor DNA was extracted from the area of vivid tumor cells in the FFPE tissue by laser microdissection (A). Fractional abundance (FA) of mutated BRAF V600E was calculated as copies of mutated DNA/(copies of mutated DNA + wildtype DNA) (B). Scale bar A: 200 μm. Figure S3. Calculation of growth rate value in NGT41 and U87MG after combination treatment. Dose response curves on relative cell count showed marked response to BRAF and/or MEK inhibitor treatment in NGT41 (A), but minimal reduction in U87MG (B). Figure S4. BRAF and MEK inhibitor induced greater apoptosis and G0/G1 arrest in the NGT41 cell line. In BRAF V600E-mutant cell lines, each treatment significantly increased the number of apoptotic cells (n = 3, *p < 0.05, **p < 0.01; Two-way ANOVA) (A). G0/G1 arrest was induced by each treatment in BRAF V600E mutant-cell lines, whereas no response was observed in BRAF-wildtype cell lines (n = 3) (B). Figure S5. Effect of BRAF and MEK inhibitor in the intracranial model. pMEK and pERK were markedly suppressed in the treatment group (A). Serial body weight calculations in the treatment group were virtually the same as in the control group (B). Histological appearance of intracranial tumor in the treatment group at endpoint was similar to that of the control group (C). Scale bar C: 50 μm. Figure S6. Analysis of the TCGA database included in R2: Genomics Analysis and Visualization Platform showed that BRAF mutations were significantly correlated with CDKN2A alterations (p = 0.025) (A) and TERT promoter mutations (p = 7.03e-03) (B). (ZIP 8066 kb) [file 40478_2019_774_MOESM2_ESM.zip › Additional file-2-Fig.S2.tif]

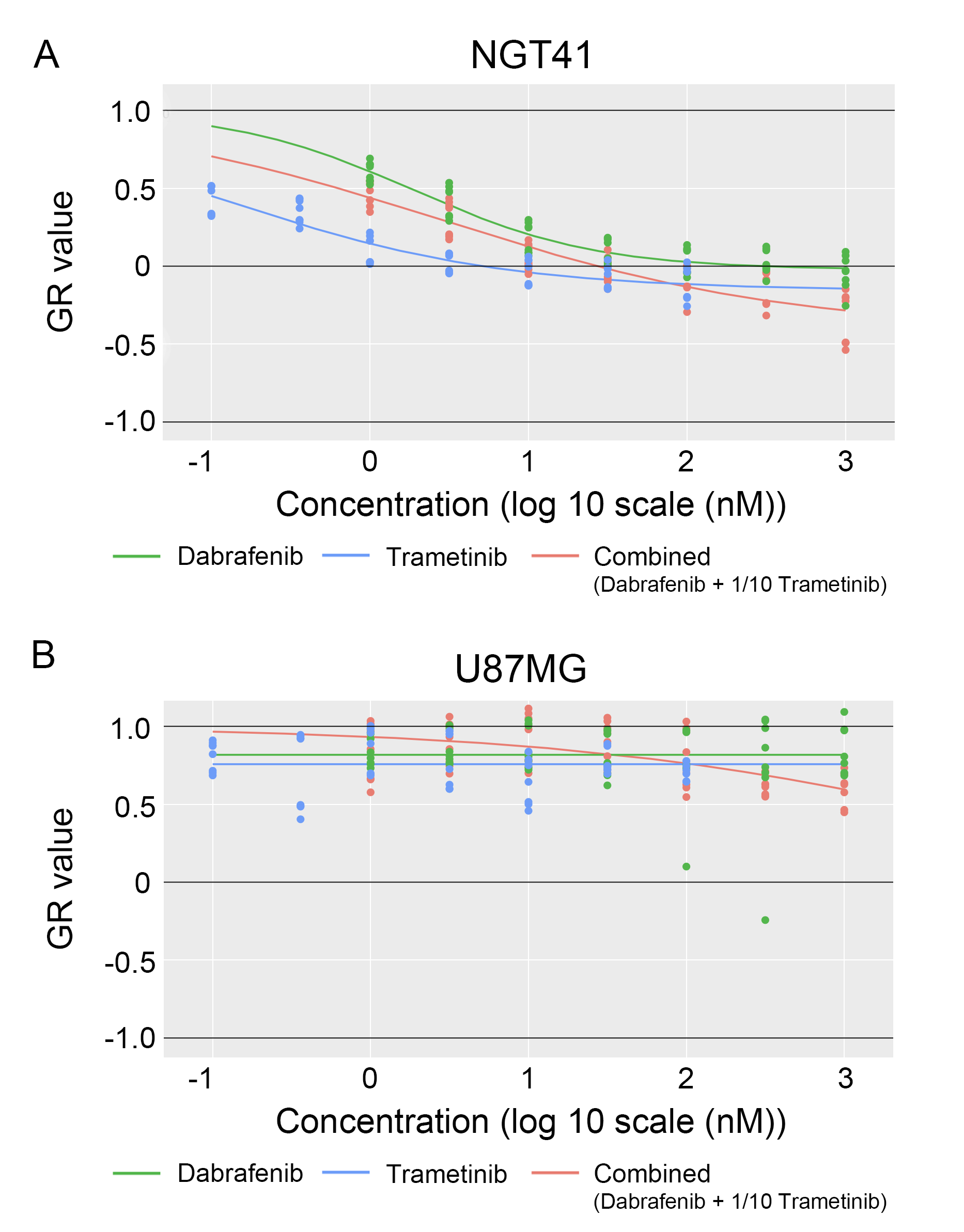

Supplement: Supplementary file 2 — Figure S1. Genetic profiles of surgical tissue and the NGT41 cell line. BRAF V600E and TERT promotor (C250T) mutation was confirmed by Sanger sequencing (A), and heterozygous loss of CDKN2A/2B was identified by the multiplex ligation-dependent probe amplification method (B). Figure S2. Evaluation of BRAF V600E using ddPCR. Tumor DNA was extracted from the area of vivid tumor cells in the FFPE tissue by laser microdissection (A). Fractional abundance (FA) of mutated BRAF V600E was calculated as copies of mutated DNA/(copies of mutated DNA + wildtype DNA) (B). Scale bar A: 200 μm. Figure S3. Calculation of growth rate value in NGT41 and U87MG after combination treatment. Dose response curves on relative cell count showed marked response to BRAF and/or MEK inhibitor treatment in NGT41 (A), but minimal reduction in U87MG (B). Figure S4. BRAF and MEK inhibitor induced greater apoptosis and G0/G1 arrest in the NGT41 cell line. In BRAF V600E-mutant cell lines, each treatment significantly increased the number of apoptotic cells (n = 3, *p < 0.05, **p < 0.01; Two-way ANOVA) (A). G0/G1 arrest was induced by each treatment in BRAF V600E mutant-cell lines, whereas no response was observed in BRAF-wildtype cell lines (n = 3) (B). Figure S5. Effect of BRAF and MEK inhibitor in the intracranial model. pMEK and pERK were markedly suppressed in the treatment group (A). Serial body weight calculations in the treatment group were virtually the same as in the control group (B). Histological appearance of intracranial tumor in the treatment group at endpoint was similar to that of the control group (C). Scale bar C: 50 μm. Figure S6. Analysis of the TCGA database included in R2: Genomics Analysis and Visualization Platform showed that BRAF mutations were significantly correlated with CDKN2A alterations (p = 0.025) (A) and TERT promoter mutations (p = 7.03e-03) (B). (ZIP 8066 kb) [file 40478_2019_774_MOESM2_ESM.zip › Additional file-2-Fig.S3.tif]

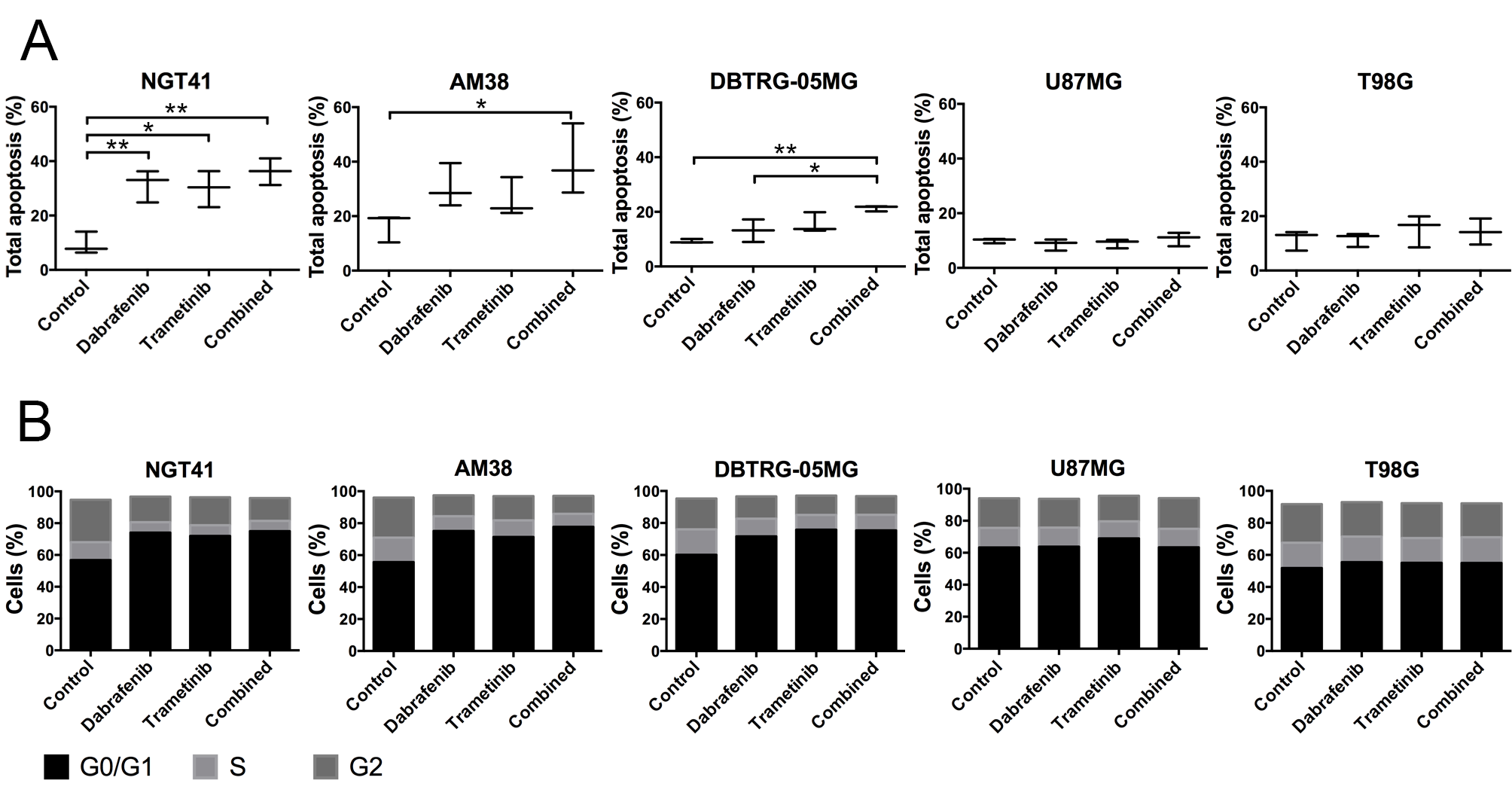

Supplement: Supplementary file 2 — Figure S1. Genetic profiles of surgical tissue and the NGT41 cell line. BRAF V600E and TERT promotor (C250T) mutation was confirmed by Sanger sequencing (A), and heterozygous loss of CDKN2A/2B was identified by the multiplex ligation-dependent probe amplification method (B). Figure S2. Evaluation of BRAF V600E using ddPCR. Tumor DNA was extracted from the area of vivid tumor cells in the FFPE tissue by laser microdissection (A). Fractional abundance (FA) of mutated BRAF V600E was calculated as copies of mutated DNA/(copies of mutated DNA + wildtype DNA) (B). Scale bar A: 200 μm. Figure S3. Calculation of growth rate value in NGT41 and U87MG after combination treatment. Dose response curves on relative cell count showed marked response to BRAF and/or MEK inhibitor treatment in NGT41 (A), but minimal reduction in U87MG (B). Figure S4. BRAF and MEK inhibitor induced greater apoptosis and G0/G1 arrest in the NGT41 cell line. In BRAF V600E-mutant cell lines, each treatment significantly increased the number of apoptotic cells (n = 3, *p < 0.05, **p < 0.01; Two-way ANOVA) (A). G0/G1 arrest was induced by each treatment in BRAF V600E mutant-cell lines, whereas no response was observed in BRAF-wildtype cell lines (n = 3) (B). Figure S5. Effect of BRAF and MEK inhibitor in the intracranial model. pMEK and pERK were markedly suppressed in the treatment group (A). Serial body weight calculations in the treatment group were virtually the same as in the control group (B). Histological appearance of intracranial tumor in the treatment group at endpoint was similar to that of the control group (C). Scale bar C: 50 μm. Figure S6. Analysis of the TCGA database included in R2: Genomics Analysis and Visualization Platform showed that BRAF mutations were significantly correlated with CDKN2A alterations (p = 0.025) (A) and TERT promoter mutations (p = 7.03e-03) (B). (ZIP 8066 kb) [file 40478_2019_774_MOESM2_ESM.zip › Additional file-2-Fig.S4.tif]

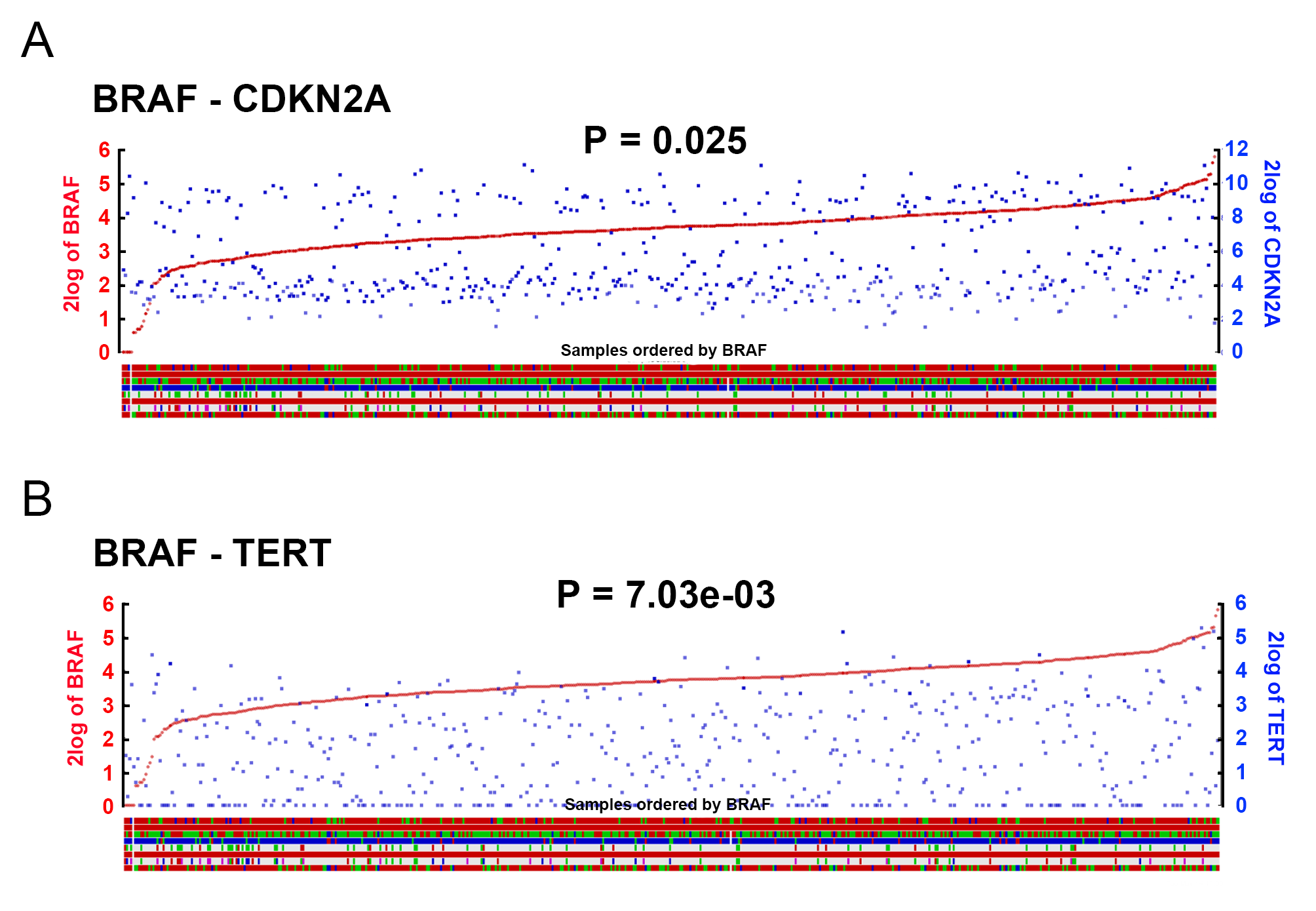

Supplement: Supplementary file 2 — Figure S1. Genetic profiles of surgical tissue and the NGT41 cell line. BRAF V600E and TERT promotor (C250T) mutation was confirmed by Sanger sequencing (A), and heterozygous loss of CDKN2A/2B was identified by the multiplex ligation-dependent probe amplification method (B). Figure S2. Evaluation of BRAF V600E using ddPCR. Tumor DNA was extracted from the area of vivid tumor cells in the FFPE tissue by laser microdissection (A). Fractional abundance (FA) of mutated BRAF V600E was calculated as copies of mutated DNA/(copies of mutated DNA + wildtype DNA) (B). Scale bar A: 200 μm. Figure S3. Calculation of growth rate value in NGT41 and U87MG after combination treatment. Dose response curves on relative cell count showed marked response to BRAF and/or MEK inhibitor treatment in NGT41 (A), but minimal reduction in U87MG (B). Figure S4. BRAF and MEK inhibitor induced greater apoptosis and G0/G1 arrest in the NGT41 cell line. In BRAF V600E-mutant cell lines, each treatment significantly increased the number of apoptotic cells (n = 3, *p < 0.05, **p < 0.01; Two-way ANOVA) (A). G0/G1 arrest was induced by each treatment in BRAF V600E mutant-cell lines, whereas no response was observed in BRAF-wildtype cell lines (n = 3) (B). Figure S5. Effect of BRAF and MEK inhibitor in the intracranial model. pMEK and pERK were markedly suppressed in the treatment group (A). Serial body weight calculations in the treatment group were virtually the same as in the control group (B). Histological appearance of intracranial tumor in the treatment group at endpoint was similar to that of the control group (C). Scale bar C: 50 μm. Figure S6. Analysis of the TCGA database included in R2: Genomics Analysis and Visualization Platform showed that BRAF mutations were significantly correlated with CDKN2A alterations (p = 0.025) (A) and TERT promoter mutations (p = 7.03e-03) (B). (ZIP 8066 kb) [file 40478_2019_774_MOESM2_ESM.zip › Additional file-2-Fig.S6.tif]
